# 2310. Shifting COVID-19 admission screening practices- An Interrupted Time Series Analysis

**DOI:** 10.1093/ofid/ofad500.1932

**Published:** 2023-11-27

**Authors:** Rossana M Rosa, Mayan Gilboa, Adriana Jimenez, David Andrews, Huy Dinh, Katiuska Parra, Octavio Martinez, Lilian M Abbo

**Affiliations:** UnityPoint Health, Urbandale, IA; Sheba Medical Center, Aventura, Florida; Jackson Health System, Infection Prevention and Control Department, Miami, FL; University of Miami, Miami, Florida; Jackson Health System, Miami, Florida; Jackson Health System, Miami, Florida; Jackson Health System/University of Miami, Miami, FL; University of Miami Miller School of Medicine, Miami Transplant Institute and Jackson Health System, Miami, FL

## Abstract

**Background:**

Screening of all patients admitted to hospitals for asymptomatic carriage of SARS-CoV-2 (SC2) using polymerase chain reaction (PCR) testing has been a widespread practice since the early months of the COVID-19 pandemic. Universal screening is an effective way of identifying carriers but can lead to delays in care and substantial financial burdens. Furthermore, hospital visitors are not screened by PCR and cannot be mandated to mask at healthcare facilities, and overall levels of COVID-19 activity community-wide have been decreasing. We describe our change in COVID-19 admission screening practices and their impact on the incidence rates of hospital-onset COVID-19 (HOC) infections.

**Methods:**

Study conducted across adult and pediatric acute care and inpatient rehabilitation hospitals at a large integrated, university affiliated, public health system in Miami, Florida. More than half of the rooms across our hospitals are semi-private. The study period was December 1, 2021 to April 30, 2023. Testing criteria on admission and masking practices are presented in Table 1. There were no visitation restrictions throughout the study period. The outcome was rates of HOC per 10,000 patient-days (PD), defined as having a test positive for SC2 on or after day 4 of admission. An Interrupted Times Series Analysis (ITSA) was done to assess changes in level or trend of rates of COVID-19 after modifying admission screening practices.

**Figure:**
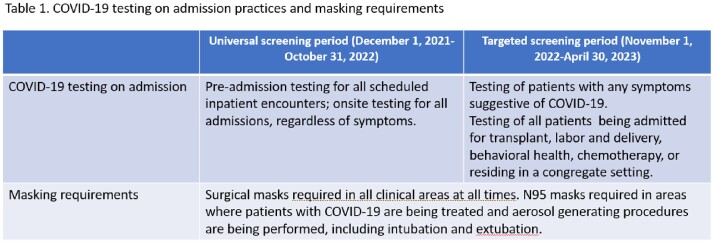

**Results:**

We identified 434 patients with HOC, with 23.5% of cases occurring in January 2022. The county-wide 7-day percent positivity was 8% when admission testing was modified, and later peaked at 22.4% in January 2023. Prior to the testing change, the HOC rate was 5.29 cases per 10,000 PD, and immediately after it was 2.74 cases per 10,000 PD. The ITSA did not show any immediate changes in level (IRR 1.50 [95% CI 0.36-6.17; p=0.57]) or in trend (IRR 1.03 [95% CI 0.73-1.46; p=0.86]) of HOC infection rates (Figure 1).

**Figure:**
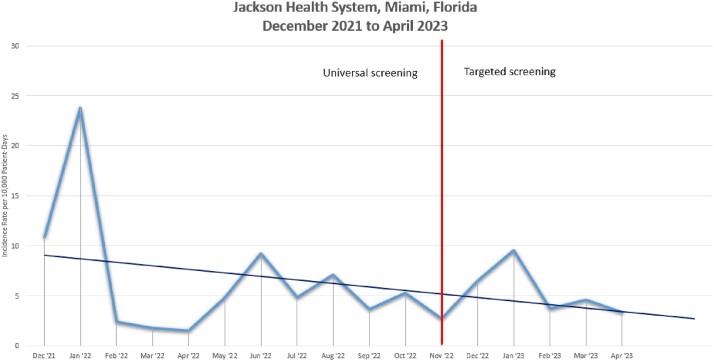

**Conclusion:**

After modifying our COVID-19 screening on admission practices to target symptomatic and high-risk patient populations we did not observe increases in the rates of HOC infections and had improved patient flows in the Emergency Department. Individual facility-level and community-wide risk assessments may need to be performed before modifying testing practices.

**Disclosures:**

**Lilian M. Abbo, MD, MBA**, Ferring: Advisor/Consultant|Pfizer: Advisor/Consultant|Regeneron: Grant/Research Support|Shionogi: Advisor/Consultant

